# Prevention of doxorubicin-induced Cardiotoxicity by pharmacological non-hypoxic myocardial preconditioning based on Docosahexaenoic Acid (DHA) and carvedilol direct antioxidant effects: study protocol for a pilot, randomized, double-blind, controlled trial (CarDHA trial)

**DOI:** 10.1186/s13063-019-3963-6

**Published:** 2020-02-04

**Authors:** Rodrigo Carrasco, María Cristina Ramirez, Kjersti Nes, Andrés Schuster, Rubén Aguayo, Marcelo Morales, Cristobal Ramos, Daniel Hasson, Camilo G. Sotomayor, Pablo Henriquez, Ignacio Cortés, Marcia Erazo, Claudio Salas, Juan G. Gormaz

**Affiliations:** 1 Cardiology Department, Clinica Alemana de Santiago, Santiago, Chile; 2 Cardiology Department, Hospital del Salvador, Santiago, Chile; 3 Cardiology Department, Hospital San Juan de Dios, Santiago, Chile; 4 Radiology Department, Clinica Alemana de Santiago, Santiago, Chile; 5 Department of Internal Medicine, University Medical Center Groningen, Groningen, The Netherlands; 6 Publich Health Institute, Faculty of Medicine, University of Chile, Santiago, Chile; 7 Medical Oncology Department, Clinica Alemana de Santiago, Vitacura 5951, Santiago, Chile

**Keywords:** CarDHA, Chemotherapy-induced cardiotoxicity, Anthracyclines, Carvedilol, DHA, Study protocol

## Abstract

**Background:**

Anthracycline-induced cardiotoxicity (AIC), a condition associated with multiple mechanisms of damage, including oxidative stress, has been associated with poor clinical outcomes. Carvedilol, a β-blocker with unique antioxidant properties, emerged as a strategy to prevent AIC, but recent trials question its effectiveness. Some evidence suggests that the antioxidant, not the β-blocker effect, could prevent related cardiotoxicity. However, carvedilol’s antioxidant effects are probably not enough to prevent cardiotoxicity manifestations in certain cases. We hypothesize that breast cancer patients taking carvedilol as well as a non-hypoxic myocardial preconditioning based on docosahexaenoic acid (DHA), an enhancer of cardiac endogenous antioxidant capacity, will develop less subclinical cardiotoxicity manifestations than patients randomized to double placebo.

**Methods/design:**

We designed a pilot, randomized controlled, two-arm clinical trial with 32 patients to evaluate the effects of non-hypoxic cardiac preconditioning (DHA) plus carvedilol on subclinical cardiotoxicity in breast cancer patients undergoing anthracycline treatment. The trial includes four co-primary endpoints: changes in left ventricular ejection fraction (LVEF) determined by cardiac magnetic resonance (CMR); changes in global longitudinal strain (GLS) determined by two-dimensional echocardiography (ECHO); elevation in serum biomarkers (hs-cTnT and NT-ProBNP); and one electrocardiographic variable (QTc interval). Secondary endpoints include other imaging, biomarkers and the occurrence of major adverse cardiac events during follow-up. The enrollment and follow-up for clinical outcomes is ongoing.

**Discussion:**

We expect a group of anthracycline-treated breast cancer patients exposed to carvedilol and non-hypoxic myocardial preconditioning with DHA to show less subclinical cardiotoxicity manifestations than a comparable group exposed to placebo.

**Trial registration:**

ISRCTN registry, ID: ISRCTN69560410. Registered on 8 June 2016.

## Background

Owing to improvements in antineoplastic treatments, overall cancer survival has improved substantially [1]. Nevertheless, these improvements have been associated with increased incidence of chemotherapy-related side effects. Anthracycline-induced cardiotoxicity (AIC) is a major cause of cancer survivor morbidity and mortality [2–4], particularly when patients develop heart failure (HF) [5]. AIC-induced HF outcomes are worse than other forms of HF and response to conventional therapy can be lower, especially in cases of late detection [6], with a 2-year mortality rate of up to 50% [7]. This contrasts with positive cancer therapy results where, for example, breast cancer mortality has dropped to sit near 10% today [8, 9].

As AIC has been historically associated with left ventricular systolic dysfunction (LVSD), the incidence of AIC has been mainly expressed as a decline in different quantitative left ventricular ejection fraction (LVEF) criteria [3, 10]. AIC-induced clinical LVSD incidence ranges around 9%, where 98% of cases develop during the first 12 months (median time 3.5 months) [6], and subclinical manifestations can reach 27% in 5 years [11]. However, this does not mean that AIC is not associated with other pathological manifestations, such as arrhythmias and biomarker elevation, where incidents of > 12% and 30–35% have been reported, respectively [12].

Evidence has shown that mitochondrial-generated reactive oxygen species (ROS) have a key role in the development and progression of AIC [13–17]. Doxorubicin tends to accumulate in cardiac mitochondria [18]. Inhibition of the enzyme topoisomerase 2β (Top2β) by doxorubicin would be the initiating event of mitochondrial dysfunction with the subsequent generation of ROS, promoting apoptosis and cardiac remodeling, key events in the development of AIC [14, 19].

The role of oxidative stress in AIC has encouraged the evaluation of several direct antioxidant strategies, without satisfactory results except in the case of carvedilol [20]. This β-blocker has uncommon ROS-suppressive properties [21], showing mixed results in trials [22–26] and observational studies [27]. Recently, a clinical trial with carvedilol in 200 patients (CECCY trial) failed to prevent a ≥ 10% reduction in LVEF at 6 months [26]. Nevertheless, the carvedilol group showed a reduction in the increments and peak levels of serum troponin I (TnI), trends towards a lesser increase in left ventricular diastolic diameter and a reduction in the percentage of patients with diastolic dysfunction.

Although β-blocker action cannot be ruled out as playing a role in AIC prevention, the cardioprotective effect from carvedilol is reported to be caused by its antioxidant properties [28–31]. A recent meta-analysis, designed to evaluate efficacy of β-blockers for primary prevention of anthracycline-derived LVSD, also suggested that the antioxidant properties of certain β-blockers could explain the efficacy observed in some trials [32]. As traditional orally administered ROS scavengers have failed to prevent AIC [20], it can be hypothesized that carvedilol advantages in AIC prevention over other potential orally administered antioxidants, may be due to carvedilol’s capacity to reach higher concentrations in cardiac cells [30].

Different factors could explain the variability of carvedilol efficacy in studies. At a mechanistic level, if the cardioprotection was hypothetically given only due to an antioxidant non-specific mechanism [33], a greater individual variability response could be expected than if the effect were a product of a specific β-blocking interaction. At a methodological level, study weaknesses, such as being observational, open-labeled, single-blind, combining cardiovascular drugs, or having a small sample size probably favored mixed results [26, 34]. High heterogeneity of studies with regard to an accumulated dose of anthracycline, cardiovascular risk factor profile, chemotherapy protocols and the involved population, as well as a high individual variability in anthracycline bioavailability and metabolism [35–37] can also be involved. The use of LVSD event based on echocardiographic consensus criterion (post-chemotherapy LVEF decline ≥ 10% to < 50%) as an AIC event to define the primary endpoint, could also be related with inconsistency in results. As several of the latter factors determine AIC incidences, they could partly explain different carvedilol efficacies among protocols, because it is expected that populations with higher AIC incidences have been more prone to benefit from cardioprotection. For example, the CECCY trial, despite its robustness, presented two methodological aspects that together make it difficult to appreciate carvedilol-induced LVSD attenuation: (1) having defined a cardiotoxicity event as a decline in LVEF ≥ 10% through the use of echocardiography (ECHO); and (2) having an event incidence lower than the originally estimated. The use of cardiac magnetic resonance (CMR) instead of ECHO plus a redefinition of the cardiotoxicity event to a drop of less than 10% of the LVEF probably would have allowed CECCY trial to appreciate carvedilol effects at cardiac function level.

Substances with indirect antioxidant properties, both by reducing cellular ROS production (such as iron chelators) and by increasing endogenous antioxidant capacity (such as cardiac preconditioning), are an option to be assessed for primary AIC prevention. However, owing to the nature of the mechanisms involved, these therapies are not exempt of risk. Dexrazoxane, an iron chelator has shown cardioprotective effects [38], nevertheless, it may cause myelosuppression and potential inhibition of doxorubicin antineoplastic efficacy [39–41]. Cardiac ischemic preconditioning has a broad preclinical base in cardiology, but it is usually complex to implement in cancer patients and its efficacy would likely be limited. To our knowledge, currently only one study is evaluating this type of strategy in AIC prevention [42].

Exercise as cardiac non-ischemic preconditioning has been proposed [43–45], but due to practical complexities for cancer patients, it is currently being tested in only one clinical trial with a suitable population [46]. Drug-based non-ischemic preconditioning has not been previously reported in AIC prevention clinical trials. A potential benefit of these interventions has been suggested by Serini et al., hypothesizing that omega-3 long-chain polyunsaturated fatty acids (EPA and DHA) could serve as cardio-protectors [47]. Cumulative evidence is not conclusive regarding the efficacy of EPA and DHA in primary, secondary and tertiary cardiovascular prevention [48, 49]. However, certain short-term interventions using high DHA doses reported attenuation of post-operative atrial fibrillation [50–52]. These effects would not be associated to omega-3 classical anti-inflammatory and antiplatelet mechanisms [53, 54], instead being based on indirect antioxidant properties [55]. Integration of DHA into cardiomyocytes induces a moderate peroxidation, weak to cause harm, though enough to activate the ROS-sensitive transcription factor Nrf2 which up-regulates antioxidant enzymes (non-hypoxic myocardial preconditioning) [56, 57]. Preclinical studies also reported the ability of n-3 long-chain fatty acids to prevent doxorubicin-induced ROS production and subsequent mitochondrial damage [57, 58].

Clinical evidence has reported DHA to be safe in metastatic breast cancer patients treated with doxorubicin [59]. Additionally, we performed a three-arm pilot protocol in patients with localized breast cancer, treated with adjuvant doxorubicin: one group with a DHA-enriched formula; another with carvedilol; and a double placebo group. Eleven patients were exposed to 2 g per day of EPA + DHA from 7 days before to 7 days after the initial chemotherapy cycle, without showing any side effects associated with the formula [60]. The DHA-enriched formula inhibited NT-proB-type natriuretic peptide (NT-ProBNP) plasmatic elevation after doxorubicin chemotherapy (48 h), suggesting a subclinical cardiotoxicity attenuation and showed a trend towards a lower LVEF decline at 10–12 months when compared to the placebo group. The lack of statistical significance was probably due to the small sample size and the limitations associated with echocardiographic sensitivity. The eleven patients exposed to 12.5 mg carvedilol every 12 h showed a significant reduction in LVEF drop at 10–12 months (51% less drop), compared to the placebo group. Unexpectedly, in this study arm, carvedilol did not impact the levels of NT-ProBNP. The study population was older and had at least one major cardiotoxicity risk factor.

In the present study, we hypothesized that breast cancer patients treated with anthracycline, preconditioned with DHA and carvedilol a week before the first chemotherapy cycle and for 90 days afterwards, will have less subclinical AIC, compared to comparable patients exposed to double placebo. We consider as subclinical AIC any manifestation of cardiac injury, such as: decrease of left ventricular function by a drop of LVEF by CMR, a decline in global longitudinal strain by two-dimensional ECHO, elevation of biomarkers or electrocardiographic alterations.

## Methods/design

### Trial design

CarDHA (Carvedilol-DHA trial) is a small, academic, randomized, double-blind, placebo-controlled, two-arm clinical trial created to test the utility of a non-hypoxic cardiac preconditioning intervention and carvedilol to prevent or attenuate subclinical manifestations of AIC.

It is supported by the Chilean National Commission for Scientific and Technological Research (CONICYT) and Clínica Alemana de Santiago. The study was initiated at San Juan de Dios Hospital in Santiago. Trial organization, management, data collection and analysis will be coordinated with clinical study staff at both centers. The co-authors have analyzed the study and agreed on this manuscript.

### Study population – patient selection and eligibility criteria

This study includes patients aged 18 to 75 years with localized breast cancer receiving chemotherapy at the oncology unit of San Juan de Dios Hospital. Specifically, patients with adjuvant and/or neoadjuvant systemic treatment with anthracycline-containing chemotherapy are included. At stages 1 and 2, verification by physical examination to exclude suspicion of metastasis is required. At stage 3, mammography/ultrasound or clinical examination via imaging staging with whole-body positron emission tomography- computed tomography (PET-CT) or abdominal thorax and pelvis CT with a bone scintigraphy is also required. Selection is open to all patients at the hospital who wish to participate and meet the eligibility criteria.

Inclusion criteria:

1. Women aged 18 to 75 years
2. Breast cancer diagnosis
3. Entering first cycle of chemotherapy
4. Performance status of 0–2 in the Eastern Cooperative Oncology Group (ECOG) score
5. Subject must be willing and able to sign an informed consent

Exclusion criteria:

1. History of renal (serum creatinine greater than 2.0 mg/ml) or hepatic insufficiency (bilirubin> 3.0 mg/dl or serum albumin < 3.5 g/dl or prothrombin time < 60% in the absence of orally administered anticoagulant therapy or ultrasound signs of chronic liver damage)
2. History of heart failure
3. History of cardiac valvulopathy
4. Baseline LVEF < 50% determined by transthoracic echocardiogram
5. Cardiogenic shock
6. Any serious medical comorbidity that determines life expectancy as < 6 months
7. Current participation in any other clinical investigation
8. Any condition that contraindicates chemotherapy (i.e., pregnancy, lactation)
9. History of severe adverse reaction to carvedilol
10. History of severe adverse reaction to DHA
11. Previous treatment with β-blockers within the last 3 months
12. Use of vitamin E, vitamin C or probucol, during the last 3 months
13. Use of orally administered anticoagulants
14. History of coagulation disorders

### Enrollment and baseline phase assessments

Patients diagnosed with localized breast cancer at the clinical campus are contacted by the study staff. After an initial clinical evaluation and explanation of the study protocol, patients agreeing to participate sign the informed consent followed by undergoing a baseline analysis including clinical evaluation and baseline laboratory and cardiovascular imaging to determine study eligibility. Baseline analyses are performed at least 10 days before the initial chemotherapy cycle, consisting of: (1) general laboratory testing, including blood cell count, INR/TTPA, full-biochemistry profile including serum creatinine and electrolytes; (2) plasma levels of NT-ProBNP and high-sensitivity cardiac troponin T (hs-cTnT); (3) resting electrocardiogram; (4) transthoracic echocardiogram; and (5) cardiac magnetic resonance. Breast cancer patients meeting the inclusion criteria are randomly allocated at a 1:1 ratio to either the intervention (DHA and carvedilol) or the control (double-placebo) arms. Randomization will be computer generated with homogeneous blocks of four by a specialist who is not related to the research group. The same person also assigns treatment and prepares the respective pillboxes with ad-hoc labeling and perform monitoring adherence. Patients, study staff, other care providers and data analysts are blinded to group allocation. The acquisition of the echocardiographic images will be performed by the same operator and will be reviewed by two other independent operators.

### Intervention

The study group is exposed to DHA at a dose of 1500 mg/day orally (preconditioning dose), starting 7 days before the beginning of the first chemotherapy cycle, and carvedilol in a dose of 12.5 mg every 12 h orally (preconditioning dose), starting 2 days before the chemotherapy cycle. The daily doses of the preconditioning regimen are delivered 7 days before the first cycle of chemotherapy in a single, weekly, red pillbox with separate compartments per day. On the day of chemotherapy, after the recovery of the red pillbox, the second weekly pillbox (green) with separate compartments per day is delivered. Both preconditioning doses will last up to 2 days after the initial cycle. From day 3 after the first chemotherapy cycle until the seventh day (green pillbox) the doses of DHA and carvedilol will be reduced to 500 mg per day and 6.25 mg every 12 h, respectively (maintenance doses). Seven days after the first chemotherapy cycle, the green pillbox is recovered and three transparent pillboxes with separate compartments per day are delivered with maintenance doses. At the end of the 3 weeks, the transparent pillboxes are recovered and three new white pillboxes are delivered with maintenance doses. This reception and delivery of new pillboxes every 3 weeks is replicated until the end of the intervention. The control arm group will likewise be exposed to double placebo. This timing follows the standard breast cancer adjuvant protocol used by the Chilean public health system, based on four anthracycline cycles each, separated by 21 days. The addition of trastuzumab or any other oncologic therapy during the standard adjuvant protocol will not modify the intervention. The pharmacological protocol will be discontinued in any patient who develops congestive heart failure or any other adverse acute cardiac effect reverting to standard care and will be unblinded. Other adverse events will be monitored and reported during the trial in each clinical evaluation by a team physician as well as in the usual oncological controls by physicians who are not part of the research team. Recommendations for Interventional Trials (SPIRIT) checklist for this protocol is also provided (Additional file 1). Generic carvedilol and its placebos were purchased specifically for this study. Natrol® DHA Super Strength and was supplied free of charge by Nutrimarket Chile, a company not participating in the study.

### Follow-up protocol

Patients participate actively in this study for 6 months, at which point the second CMR is performed. One year after the first cycle of chemotherapy, a final phone contact is to be carried out.

Follow up is performed and monitored by trained study personnel who interview the patients by phone or in person, including:

1. Clinical follow-up at 30, 90 and 180 days
2. General laboratory analyses: blood cell count and full-biochemistry profile, including creatinine and electrolytes, 2 and 4 days after the initial chemotherapy cycle
3. NT-ProBNP and hs-cTnT plasma levels (by ELISA) 2, 4 and 90 days after the first chemotherapy cycle
4. Oxidative stress parameters analysis at days 2, 4 and 90 after the first chemotherapy cycle
5. Resting electrocardiogram 2, 4 and 90 days after the first chemotherapy cycle
6. Transthoracic echocardiogram at 90 and 180 days after the chemotherapy cycle
7. CMR at 180 days after the initial chemotherapy cycle
8. Final phone contact 1 year after the first chemotherapy cycle

### Study objectives – primary and secondary endpoints

The trial includes four co-primary endpoints to assess the efficacy of the proposed strategy to inhibit or attenuate the subclinical manifestations of anthracycline-induced cardiotoxicity (AIC). The co-primary endpoints include two imaging variables, a serum biomarker and electrocardiographic variables. The first co-primary endpoint is changes in LVEF from baseline control to 180 days after the first chemotherapy cycle, determined by CMR. The second co-primary endpoint is the percentage of changes in global longitudinal strain (GLS) comparing baseline with 90 and 180 days after the first cycle, determined by two-dimensional ECHO. The third co-primary endpoint is the elevation in serum biomarkers, including NT-ProBNP between baseline and 48 h after the first chemotherapy cycle and/or elevations in levels of hs-cTnT at 2, 4 and 90 days. The fourth co-primary endpoint is the prolongation in the corrected QT interval, 48 h after the first chemotherapy cycle.

Additionally, three secondary endpoints were included to determine other imaging and clinical variables, as well as to evaluate systemic biomarkers of oxidative stress. The first secondary endpoint is the occurrence of major adverse cardiac events during follow-up such as: cardiac death, acute coronary syndrome, acute pulmonary edema, clinical manifestations of heart failure and life-threatening arrhythmias. The second secondary endpoint is changes in LVEF from baseline compared to 90 and 180 days after the first chemotherapy cycle, determined by two-dimensional ECHO. The third secondary endpoints are biomarkers of oxidative stress damage, as well as parameters of intracellular and extracellular antioxidant balance. In order to evaluate the oxidative stress damage, plasma lipoperoxidation levels (malondialdehyde levels) will be measured at 2, 4 and 180 days after the initial chemotherapy cycle. To evaluate antioxidant balance, the Erythrocyte Thiol Index (GSH/GSSG) will be determined at 2, 4 and 90 days after the first chemotherapy cycle. Given this is a pilot study, outcomes will include the total number of patients screened, those eligible, those providing informed consent and those completing the intervention treatment.

### Timeline for participants

**Fig. 1:**
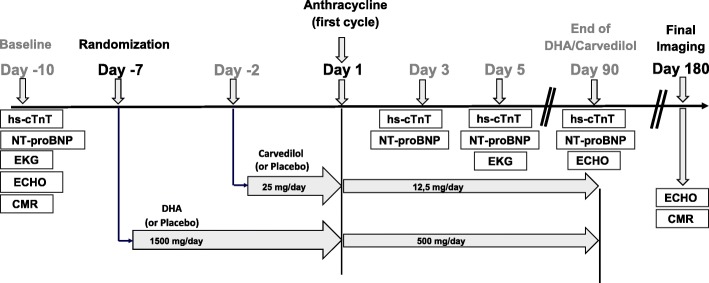
Timeline of the Carvedilol-DHA (CarDHA) trial. *hs-cTnT* high-sensitivity cardiac troponin T, *NT-ProBNP* NT-proB-type natriuretic peptide, *EKG* resting electrocardiogram, *ECHO* echocardiography, *CMR* cardiac magnetic resonance, *DHA* docosahexaenoic acid

**Fig. 2:**
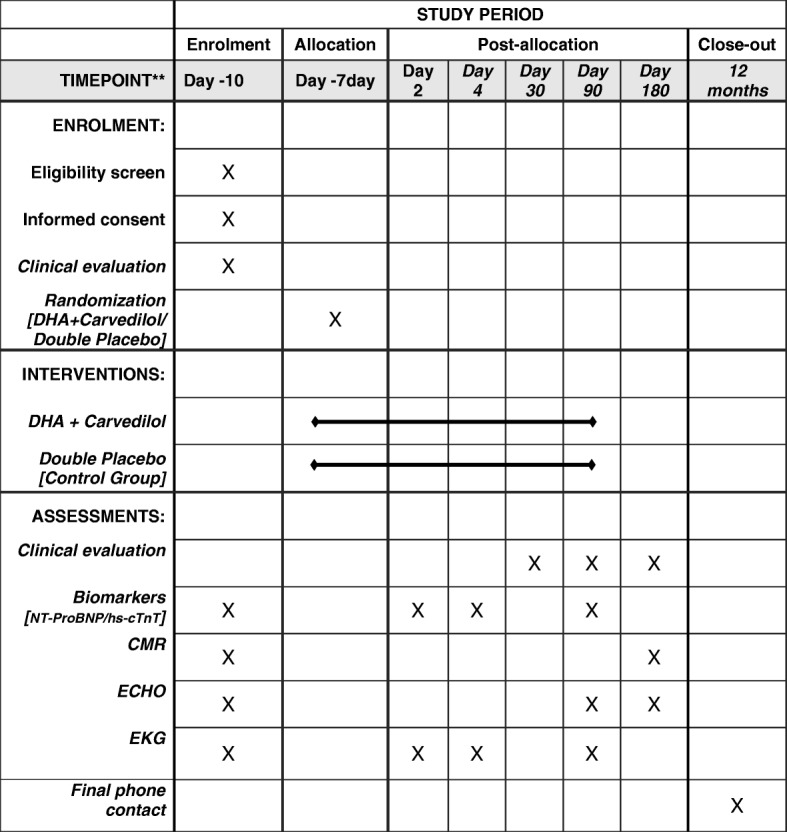
Standard Protocol Items: Recommendation for Interventional Trials (SPIRIT) Figure (required for study protocols). *CMR* cardiac magnetic resonance, *DHA* docosahexaenoic acid, *ECHO* echocardiography, *hs-cTnT* high-sensitivity cardiac troponin T, *NT-ProBNP* NT-proB-type natriuretic peptide, *EKG* resting electrocardiogram

Figure 1 represents the patient timeline. Outlined in Fig. 2 is the study schedule of enrollment and assessments (Standard Protocol Items: Recommendations for Interventional Trials (SPIRIT Figure).

### Sample size and data analysis

The sample size was estimated on the basis of results from our previous protocol (63), where the carvedilol-supplemented group showed a 51% reduction of differential LVEF (ΔLVEF) compared with the placebo group. ΔLVEF was determined between the last follow-up ECHO at approximately 1 year (10–12 months) with respect to baseline ECHO, both performed by two-dimensional evaluation. Assuming normal distribution and a common relative standard deviation of 48% (supplemented group), with 5% two-sided significance, a sample size of 32 will be required to provide 80% statistical power to detect a 12.5% ΔLVEF reduction assessed by CMR to provide greater precision.

Results will be analyzed according to intention-to-treat criteria, including all randomized patients who are able to initiate intervention, independent of treatment and follow-up period durations. Continuous variables will be expressed as means ± standard deviation or medians and interquartile ranges depending on their distribution. Distribution will be analyzed using the Kolmogorov-Smirnov test. Student’s *T* test and Pearson correlation analyses will be applied for normally distributed continuous variables. The Mann-Whitney *U* test and Spearman’s correlation will be used for non-normally distributed continuous variables. Categorical variables will be expressed as numbers and frequencies (%). Differences between the frequencies will be performed by the chi-square test or by Fisher’s exact test. Multiple linear regression analysis will be used to assess independent predictors of the absolute semiannual changes in ΔLVEF, to identify factors associated with ΔLVEF. The difference between the groups will be considered significant with a *p* value of < 0.05.

## Discussion

CarDHA is a limited, academic, randomized, placebo-controlled, double-blind, two-arm clinical trial designed to evaluate the efficacy of the proposed strategy to inhibit or attenuate subclinical manifestations of anthracycline-induced cardiotoxicity (AIC).

The four co-primary endpoints of the study are: (1) changes in LVEF as evidenced by CMR; (2) changes in global longitudinal strain (GLS) manifested in echocardiographic imaging; (3) elevations in levels of serum biomarkers (NT-ProBNP/hs-cTnT); and (4) QT interval prolongation by electrocardiographic evaluation.

Unlike similar AIC primary prevention protocols based on carvedilol alone, to our knowledge this trial is the first cardio-oncology study to: (1) be expressly designed as a therapeutic strategy focusing on attenuating the oxidative stress associated to AIC, rather than on the β-blocking effects of carvedilol; and (2) attempt to evaluate the potential cardioprotective effects of a dual strategy (comprising non-ischemic cardiac preconditioning through endogenous antioxidant capacity enhancement through DHA, plus the direct antioxidant properties of carvedilol, in a sequential regimen specifically designed to attenuate chemotherapy-induced oxidative stress). We expect the inhibition or attenuation of the anthracycline-derived oxidative heart injury generated by two unrelated antioxidant pathways will be more efficient than the potential of one the pathways alone.

The main limitations of our study are the small sample size, the non-use of consensus criterion (post-chemotherapy LVEF decline ≥ 10% to < 50%) to define a cardiotoxic event for the first co-primary endpoint [6] and to have a single-center trial. The use of CMR imaging to evaluate our first co-primary endpoints, the gold-standard method to evaluate LVEF decline, partially compensates the first two limitations, since we are not limited by echocardiographic sensitivity and should be able to detect subclinical cardiotoxicity at the early stages [10]. Additionally, although most cardio-oncology trials have used the ≥ 10% consensus criterion to establish cardiotoxicity, that range was defined arbitrarily as a criterion for two reasons: first, it is widely used as cut-off point to evaluate the continuity of oncological treatment; and second, it reflects the echocardiographic sensitivity limits to establish a decline in LVEF with certainty [10]. A smaller than 10% in AIC-induced LVEF reduction does not imply the absence of cardiotoxicity. Thus, other clinical trials with relatively small sample sizes using CMR to evaluate LVSD as the primary endpoint, such as the PRADA and MANTICORE trials, also rule out the criterion of a LVEF decline ≥ 10% for event definition, in favor of comparing mean differences between the groups [61, 62]. Moreover, limiting the description of a cardiotoxic event to a consensus definition of decline in LVEF can underestimate the manifestation of other types of cardiotoxicity (such as an elevation in cardiac biomarkers), some of which could be associated with the future development of later clinical manifestations [63, 64]. Regarding the lack of representativeness associated with single-center studies, the San Juan de Dios is a public hospital that provide healthcare to a large urban population, and several rural surrounding locations. It also has the advantage that all echocardiographic images will be performed by the same person, and analyzed by the same two specialist. Additionally, it plays in our favor the fact that in Chile the doxorubicin protocol for localized breast cancer is standardized in all public hospitals and is quite similar among private health services.

The relative short follow-up time (6 months) could also be considered a problem in this study. Nevertheless, Cardinale et al. previously reported in a large prospective study, including 2625 patients, an early LVEF drop in patients developing cardiotoxicity [6]. In that study the median time elapsed between the end of chemotherapy and cardiotoxicity development was 3.5 months. Therefore, it sems to be difficult not to find differences at 6 months through CMR. Finally, for some authors, the use of a combination therapy could also be considered a limitation [35]. This implies a difficulty when comparing our results to similar studies based on carvedilol alone. Our design also difficult estimating attributable fraction to carvedilol if we find beneficial effects. Notwithstanding, we maintain that beyond the use of CMR, the greatest contribution of this protocol is to evaluate a prophylactic strategy completely designed to attenuate the oxidative stress derived from AIC, with the inclusion of two different antioxidant strategies administered sequentially to enhance the effects.

In conclusion, CarDHA is the first randomized trial designed to evaluate the potential cardioprotective effects of a dual antioxidant strategy comprising a non-ischemic pharmacologic cardiac preconditioning based on DHA and carvedilol, specifically designed to attenuate oxidative stress as a key factor in AIC development and progression. Potentially favorable results of this study will generate the basis for larger clinical trials to further explore the efficacy of this innovative strategy.

## Trial status

The protocol was reviewed and approved by the Institutional Review Board of the San Juan de Dios Hospital and the Ethics Committee of the University of Chile, whose medical school is associated with San Juan de Dios Hospital, as only the university has a committee accredited by the Chilean Ministry of Health.

**Fig. 3:**
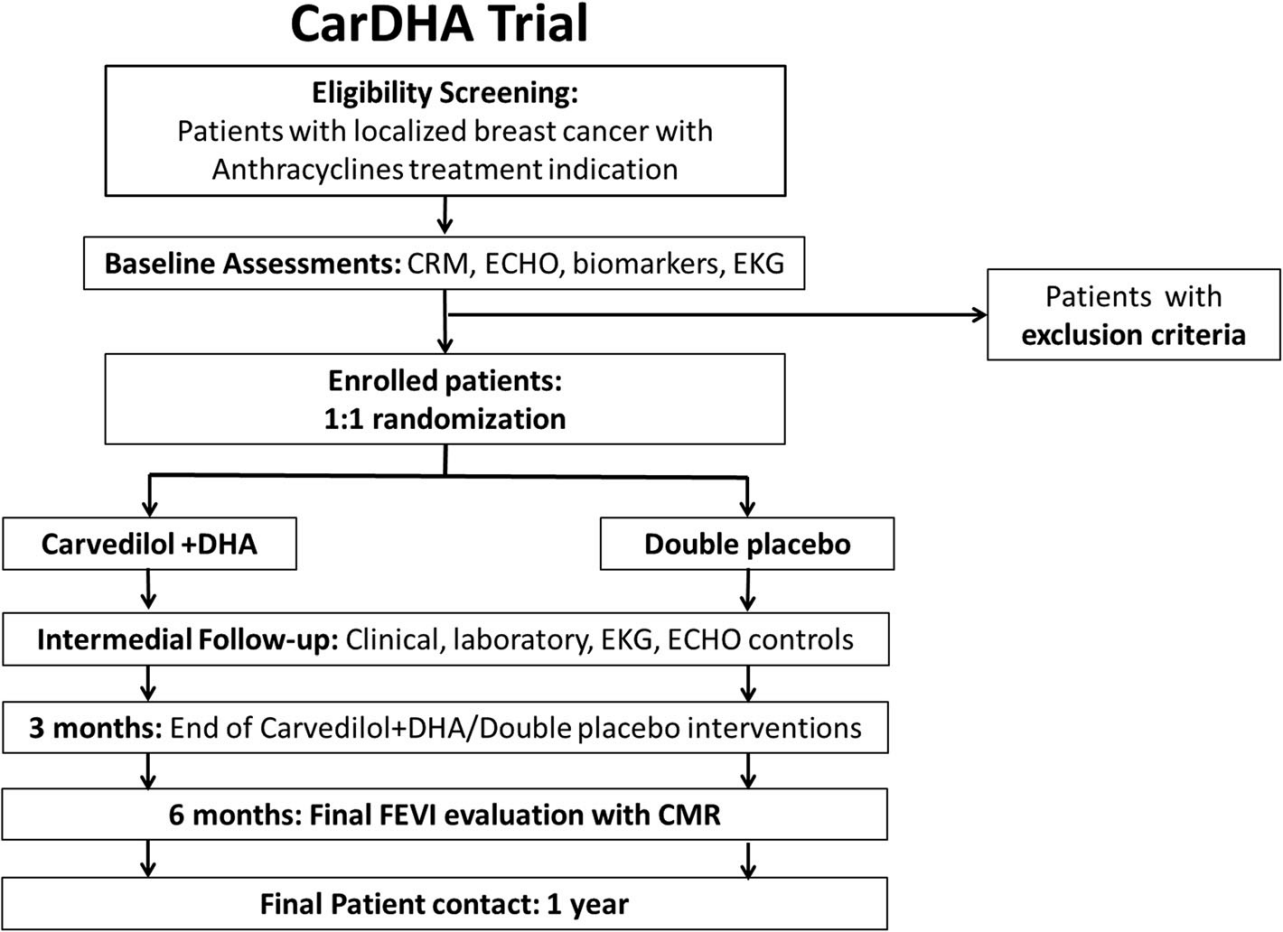
Flow chart of the Carvedilol-DHA (CarDHA) trial

Enrollment was started on 30 May 2016. However, the first patient entered into the study on 10 August 2016. As of 26 October 2018, the study had enrolled 30 patients at the San Juan de Dios Hospital. The study is being conducted in accordance with “Good Clinical Practice” recommendations, based on the Declaration of Helsinki (2002). The trial has been registered at the ISRCTN registry with the code ISRCTN69560410 applied on 8 June 2016. A flow chart of this study is presented in Fig. 3.

## Supplementary information

**Additional file 1.** Standard Protocol Items: Recommendations for Interventional Trials (SPIRIT) 2013 Checklist: recommended items to address in a clinical trial protocol and related documents.

## Data Availability

The datasets generated and/or analyzed during the current study are available from the corresponding author on reasonable request.

## Abbreviations

AIC: Anthracycline-induced cardiotoxicity
CarDHA: Carvedilol-DHA trial
CMR: Cardiac magnetic resonance
DHA: Docosahexaenoic acid
ECHO: Echocardiography
GLS: Global longitudinal strain
HF: Heart failure
hs-cTnT: High-sensitivity cardiac troponin T
LVEF: Left ventricular ejection fraction
LVSD: Left ventricular systolic dysfunction
NT-ProBNP: NT-proB-type natriuretic peptide
ROS: Reactive oxygen species
TnI: Troponin I
Top2β: Topoisomerase 2 Beta
ΔLVEF: Differential LVEF
